# Biogenic Preparation and Characterization of Silver Nanoparticles from Seed Kernel of *Mangifera indica* and Their Antibacterial Potential against *Shigella* spp.

**DOI:** 10.3390/molecules28062468

**Published:** 2023-03-08

**Authors:** Sudha Angamuthu, Selvankumar Thangaswamy, Amutha Raju, Fohad Mabood Husain, Bilal Ahmed, Nasser A. Al-Shabib, Mohammed Jamal Hakeem, Syed Ali Shahzad, Saud A. Abudujayn, Suliman Y. Alomar

**Affiliations:** 1 Bon Secours Arts & Science College for Women, Department of Biotechnology, Sowthapuram (PO), Near Veppadai, Namakkal 638008, Tamil Nadu, India; sudha29.a@gmail.com; 2 Department of Biotechnology, Mahendra Arts and Science College (Autonomous), Namakkal 637501, Tamil Nadu, India; selvankumar75@gmail.com; 3 Centre for Post Graduate and Research Studies, Periyar University, Salem 636001, Tamil Nadu, India; drramutha@rocketmail.com; 4 Department of Food Science and Nutrition, King Saud University, Riyadh 11495, Saudi Arabia; nalshabib@kau.edu.sa (N.A.A.-S.); mhakeem@ksu.edu.sa (M.J.H.); syedalishahzad@gmail.com (S.A.S.); 444105567@student.ksu.edu.sa (S.A.A.); 5 School of Chemical Engineering, Yeungnam University, Gyeongsan 38541, Republic of Korea; 6 Department of Zoology, King Saud University, Riyadh 11495, Saudi Arabia; syalomar@ksu.edu.sa

**Keywords:** AgNPs, drug resistance, enteric pathogen, shigellosis, spectral analysis

## Abstract

Shigellosis is a serious foodborne diarrheal disease caused by the *Shigella* species. It is a critical global health issue. In developing countries, shigellosis causes most of the mortality in children below 5 years of age. Globally, around 165 million cases of diarrhea caused by *Shigella* are reported, which accounts for almost 1 million deaths, in which the majority are recorded in Third World nations. In this study, silver nanoparticles were synthesized using *Mangifera indica* kernel (MK-AgNPs) seed extracts. The biosynthesized *M. indica* silver nanoparticles (MK-AgNPs) were characterized using an array of spectroscopic and microscopic tools, such as UV–Vis, scanning electron microscopy, particle size analyzer, Fourier transform infrared spectroscopy, and X-ray diffractometer. The nanoparticles were spherical in shape and the average size was found to be 42.7 nm. The MK-AgNPs exhibited remarkable antibacterial activity against antibiotic-resistant clinical *Shigella* sp. The minimum inhibitory concentration (MIC) value of the MK-AgNPs was found to be 20 μg/mL against the multi-drug-resistant strain *Shigella flexneri*. The results clearly demonstrate that MK-AgNPs prepared using *M. indica* kernel seed extract exhibited significant bactericidal action against pathogenic *Shigella* species. The biosynthesized nanoparticles from mango kernel could possibly prove therapeutically useful and effective in combating the threat of shigellosis after careful investigation of its toxicity and in vivo efficacy.

## 1. Introduction

Shigellosis is a diarrheal disease caused by the *Shigella* species and is a serious health concern globally. Mortality is high in children below 5 years of age, particularly in developing countries [1]. Furthermore, around 165 million cases of diarrhea were reported across the globe, causing approximately one million deaths annually, most of which were recorded in Third World countries [1]. Shigellosis is a burden on middle-income and low-income countries where clean drinking water, proper nutrition, sustained sanitation, and healthcare facilities are limited. Although all age groups are susceptible to *Shigella* infection, it is endemic in children below 5 years of age [2].

Dysentery caused by *Shigella flexneri* is considered to be a very common, explosive disease, and it is one of the major causes for morbidity and mortality in children in the Third World. Low infectious dose, such as 10–100 bacteria, can cause infection and lead to large outbreaks. *S. flexneri* has the unique ability to tolerate adverse conditions, such as low pH and elevated temperatures, for an extended period of time. Thus, *S. flexneri* can survive in acidic foods and tolerate the acidic conditions prevailing in the human stomach [2,3,4]. Control of *S. flexneri* in food has been mostly limited to the use of conventional sterilization techniques or addition of bactericidal substances, but these methods are not cost-effective and may show adverse effects on sensory properties and food quality [3].

Furthermore, antibiotics resistance in bacteria has become a major health concern globally [5]. In modern medicine, antibiotic treatment is extremely important to treat infections as it plays a vital role in decreasing prevalence and mortality rates associated with infectious disease [6]. However, multidrug resistance leads to an increased period of hospitalization and increased mortality [7]. A matter of great concern is the emergence of drug-resistant *Shigella* strains, which has led to treatment impasse, and treatment of diarrheal illness has become challenging [1,8]. Therefore, there is an urgent need to develop novel therapeutic agents that can arrest the growth of drug-resistant foodborne pathogens, such as *Shigella*.

Recently, targeting bacteria using nanomaterials as antibacterial agents is being exploited by researchers across the globe. Nanoparticles offer unique properties, such as increased surface-to-volume ratio and distinct energy levels [7]. Nanoparticles can be synthesized using chemical and physical methods, but these routes of nanoparticle synthesis have proved flawed in terms of particle aggregation, cost effectiveness, release of hazardous chemicals, and environmental deterioration [9]. Therefore, it is imperative to use safe, cost-effective, and eco-friendly methods of nanoparticle synthesis. In this regard, green synthesis of nanoparticles using biological template has become the most extensively recognized way of preparing metal nanoparticles. Moreover, plant extracts have been exploited for phytosynthesis of various nanoparticles, as phytocompounds present in plant extracts catalyze the biosynthesis and capping of nanoparticles [9]. Furthermore, the process is simple and rapid, and one-step reaction and synthesis can be performed at room temperature. Plant biomaterial reduces the use of hazardous chemicals and toxic solvents. Overall, the green synthesis route is a single step, economical, reproducible, and sustainable [9].

Mango (*Mangifera indica* L.) belongs to the Anacardiaceae family. Mango kernel is an extremely nutritious food [10] and contains diverse phytocompounds [11]. Several studies have reported mango seed kernels for anti-enteric [12], antibacterial [13], antidiarrheal, anti-salmonella [14], and antioxidant properties [15]. The AgNPs have been effectively biosynthesized using various parts of the mango, including the peel [16], leaves [17], the flower [18], and the kernel [19]. However, AgNPs synthesis using mango kernels for efficacy against *Shigella* species is yet to be reported. Therefore, this study is aimed at investigating the potential of the Indian traditional medicinal plant *M. indica* in the green synthesis of silver nanoparticles. Furthermore, biogenic silver nanoparticles were explored for their antimicrobial potential against multidrug-resistant (MDR) clinical isolates of *Shigella* sp.

## 2. Results and Discussion

Green synthesis of nanoparticles is a convenient, ecofriendly, and more suitable method for large-scale synthesis, especially from plants that possess high antibacterial and antioxidant capacity [18,20]. This study outlines an innovative idea of producing bioactive nanoparticles using waste material. Seeds from mango fruits are usually thrown away into the environment as waste and this fruit waste was utilized for the synthesis of silver nanoparticles in the present investigation.

**Figure 1:**
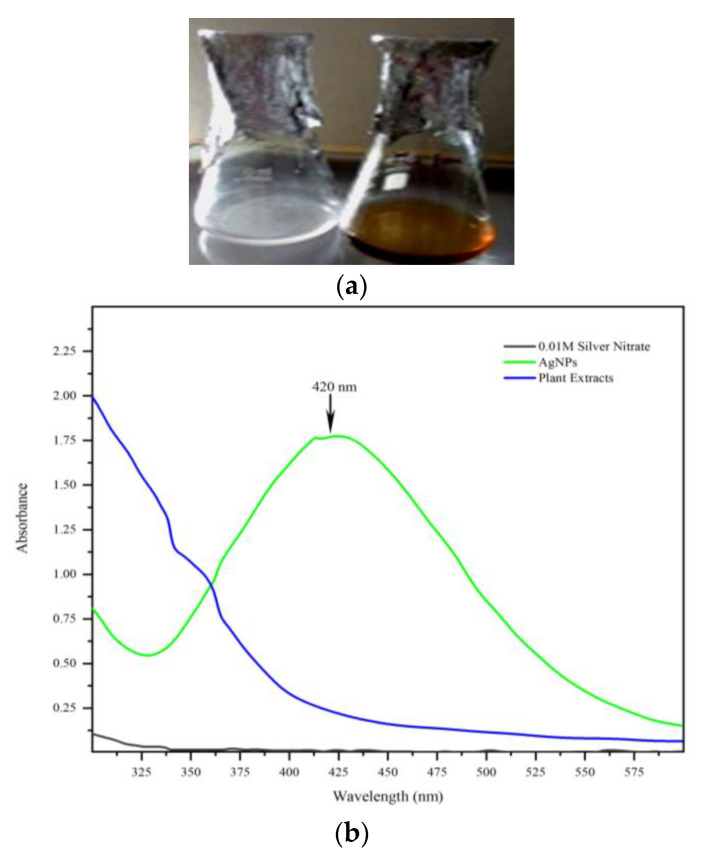
(**a**) Synthesis of MK-AgNPs from mango seed kernel ethanol extract. (**b**) UV–visible spectra of MK-AgNPs.

The visual colour alteration in the reaction mixture is the first indication of the synthesis of silver nanoparticles [21]. The addition of mango seed kernel extract to silver nitrate solution initiated the particle synthesis reaction within a few minutes and the change of colour in the reaction mixture was observed. Colourless silver nitrate solution was changed into a dark brown colour, which indicated the reduction of Ag^+^ to Ag^0^. The quick colour change of the colourless reaction mixture to brown colour indicates the rapid synthesis of silver nanoparticles, and finally, it turned to a dark, brownish-black colour (Figure 1). Similar visual observations were recorded in silver nanoparticles synthesized from *Mangifera indica aqueous* extract [22].

**Figure 2:**
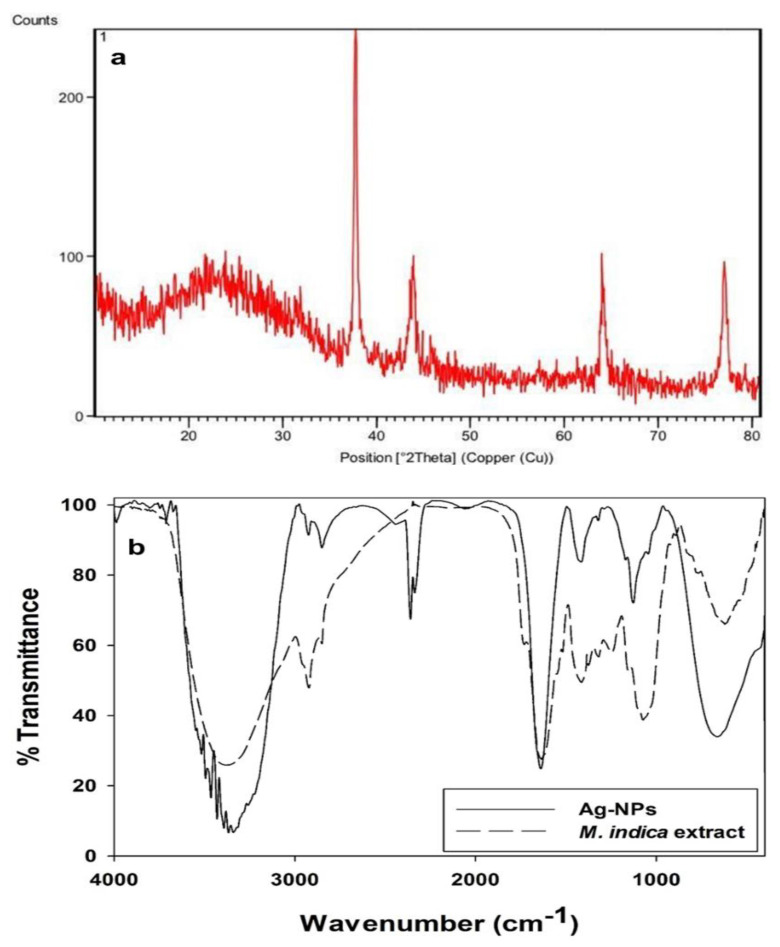
(**a**) X-ray diffraction pattern of MK-AgNPs. (**b**) FTIR Analysis of synthesized MK-AgNPs.

The colour change was analyzed using UV–Vis spectrophotometer, which confirmed the successful synthesis of nanoparticles (Figure 2). UV–Vis spectroscopy is the most precise approach for finding the presence of nanoparticles. The UV–Vis spectra of synthesized nanoparticles were recorded on a spectrophotometer in the 300–600 nm range. The MK-AgNPs exhibited their characteristic absorption maximum peak at 420 nm. This corroborates well with the findings of several other studies. AgNPs synthesized using extract of *Tectona grandis* (seed) and *Lantana camara* (leaves) showed an absorption maximum at 450 nm [16,23].

X-ray diffraction pattern displayed diffraction peaks at 2θ of 37.7°, 44.0°, 64.0°, and 77.0°, which can be indexed to (111), (200), (220), and (311) planes of face-centered cubic (FCC) of silver nanoparticles of Joint Committee on Powder Diffraction Standards (JCPDS) file No. 04-0783 (Figure 2). A similar diffraction pattern was observed by Sundeep et al. [17]. The narrow peak indicated the crystalline nature of Ag nanoparticles. Secondary metabolites of extract were analyzed by FT-IR spectrum, which showed a broad peak at 3339.69 cm^−1^ assigned to OH group stretching vibrations (Figure 2b). The peak at 3341.44 cm^−1^ indicated the presence of alcohols and phenols stretching. The band at 1639.38 cm^−1^ is assigned to the amines and N–H stretching vibration. The FT-IR analysis indicated many bioactive compounds, such as phenols, alkaloids, flavonoids, and tannin, which appear to be responsible for the synthesis, stabilization, and capping of AgNPs. Secondary metabolites, especially polyphenols in the extract, mediate the reduction of ions to the atomic form [22].

Furthermore, phytochemical analysis indicated the presence of numerous biomolecules in mango seed kernel extract (Table S1), which are responsible for the synthesis and bioactivity of nanoparticles. Donga et al. [23] reported the presence of phenols, flavonoids, tannins, and coumarins in the extract of *M. indica* seed powder, while its polar solvent methanol extract and aqueous extract revealed the presence of phenols, flavonoids, tannins, cardiac glycosides, coumarins, and anthocyanins. The presence of these important secondary metabolites may play a significant role in the formation, reduction, capping, and stabilisation of AgNPs. Furthermore, Jaffri and Ahmad [24] reported that phenolic compounds and proteins are actively involved in the reduction of silver to silver nanoparticles by *Prunus cerasifera* fruit extract. They reported the presence of functional groups, such as alcohol or phenol, carboxylic acid, ketones, amines, aromatics, aromatic amines, aliphatic amines, alkyl halides, and alkynes in the extract.

**Figure 3:**
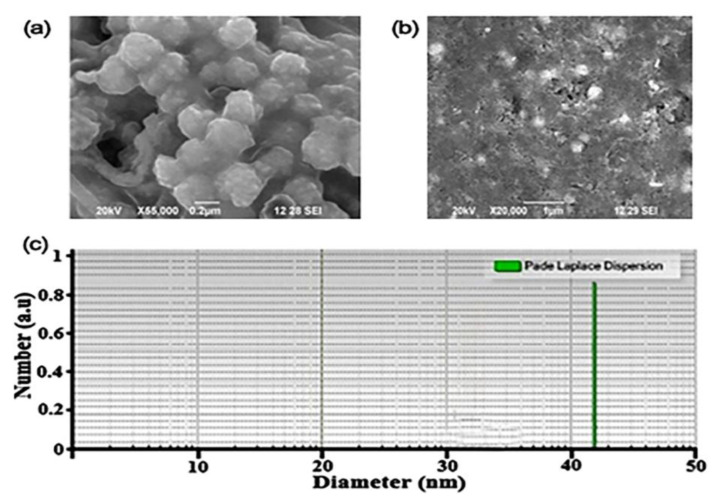
(**a**,**b**) SEM Image of MK-AgNPs. (**c**) Particle size analyzer of MK-AgNPs.

Morphology of the synthesized MK-AgNPs was determine using SEM analysis. The synthesized AgNPs were found to be spherical, and their size ranged from 40 to 170 nm (Figure 3a,b). Figure 3c shows the particle size analyzer (PSA) graph of MK-AgNPs. Mean particle size of the synthesized MK-AgNPs was found to be 42.6 nm under dynamic light scattering. Sundeep et al. [17] reported mean particle size of 31.7 nm for AgNPs synthesized using extract of mango leaves. Variations in morphology and particle sizes of the synthesized MK-AgNPs could be attributed to the diverse aggregation influence of varying extract concentrations. Moreover, different plant parts demonstrate different levels of reducibility, and variations in extract concentrations, temperature, and contact time also play a key role in determining the size of NPs. Because mango is rich in polyphenols, it acts as a capping agent, reducing the AgNO_3_ to coat the AgNPs surface. Hence, nanoparticle aggregation was due to the covering of the AgNPs by capping agents, causing them to adhere to each other. Furthermore, the nanoparticles’ sizes increased in conjunction with extract concentrations, causing changes in shape, which can be attributed to the growth rate of crystal nucleus [25].

In total, 30 diarrheal samples were processed to isolate the *Shigella* strains. A total of 13 isolates were identified in accordance with Bergey’s Manual of Systematic Bacteriology. Drug resistance pattern of clinical isolates of *Shigella* sp. is illustrated Figure S1a. All the test isolates showed highest resistance to chloramphenicol (96.6%), followed by ampicillin (94.6%) and tetracycline (96.2%). These results corroborate well with the previous reported studies [21,22,23]. In this study, comparatively higher resistance was observed to gentamicin (95.3%), which is contradictory to the findings reported by Taneja et al. [26]. These results indicate the emergence of multiple drug-resistant strains due to the unabated and improper use of antibiotics for shigellosis treatment. Among the 13 clinical isolates, four were found to demonstrate resistance to more than five groups of tested antibiotics (Figure S1b). One isolate showed resistance against third-generation cephalosporins. Hence, this isolate was identified further through 16s rRNA partial sequencing and the sequence was submitted to GENBANK.

*Shigella flexneri* strain ASSA (Accession number: MW830164.1)

Origin 1

acggtagcta ataccgcata acgtcgcaag accaaagagg gggaccttcg ggcctcttgc 61

catcggatgt gcccagatgg gattagctag taggtggggt aacggctcac ctaggcgacg 121

atccctagct ggtctgagag gatgaccagc cacactggaa ctgagacacg gtccagactc 181

ctacgggagg cagcagtggg gaatattgca caatgggcgc aagcctgatg cagccatgcc 241

gcgtgtatga agaaggcctt cgggttgtaa agtactttca gcggggagga agggagtaaa 301

gttaatacct ttgctcattg acgttacccg cagaagaagc accggctaac tccgtgccag 361

cagccgcggt aatacggagg gtgcaagcgt taatcggaat tactgggcgt aaagcgcacg 421

caggcggttt gttaagtcag atgtgaaatc cccgggctca acctgggaac tgcatctgat 481

actggcaagc ttgagtctcg tagagggggg tagaattcca ggtgtagcgg tgaaatgcgt 541

agagatctgg aggaataccg gtggcgaagg cggccccctg gacgaagact gacgctcagg 601

tgcgaaagcg tggggagcaa acaggattag ataccctggt agtccacgcc gtaaacgatg 661

tcgacttgga ggttgtgccc ttgaggcgtg gcttccggag ctaacgcgtt aagtcgaccg 721

cctggggagt acggccgcaa ggttaaaact caaatgaatt gacgggggcc cgcacaagcg 781

gtggagcatg tggtttaatt cgatgcaacg cgaagaacct tacctggtct tgacatccac 841

ggaagttttc agagatgaga atgtgccttc gggaaccgtg agacaggtgc tgcatggctg 901

tcgtcagctc gtgttgtgaa atgttgggtt aagtcccgca acgagcgcaa cccttatcct 961

ttgttgccag cggtccggcc gggaactcaa aggagactgc cagtgataaa ctggaggaag 1021

gtggggatga cgtcaagtca tcatggccct tacgaccagg gctacacacg tgctacaatg 1081

gcgcatacaa agagaagcga cctcgcgaga gcaagcggac ctcataaagt gcgtcgtagt 1141

ccggattgga gtctgcaact cgactccatg aagtcggaat cgctagtaat cgtggatcag 1201

aatgccacgg tgaatacgtt cccgggcctt gtacacaccg cccgtcacac catgggagtg 1261

ggttgcaaaa gaag

**Figure 4:**
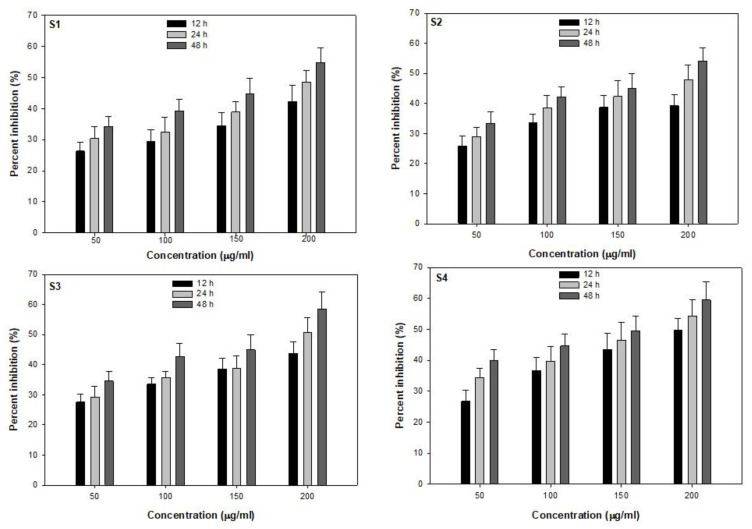
Antibacterial activity of MK-AgNPs against clinical *Shigella* isolates.

Drug resistance among bacteria is a big challenge in the treatment and management of infectious diseases, especially in pediatric shigellosis. Previous studies have reported and elaborately discussed the prevalence of drug resistance among *Shigella* sp. [6,22]. The MK-AgNPs exhibited antibacterial activity against four drug-resistant isolates of enteric pathogens *Shigella* sp. at concentrations 50, 100, 150, and 200 µg/mL (Figure 4). The recorded percentage growth inhibition of clinically important drug-resistant *Shigella* sp. (*S. flexneri*, S2, S3, and S4) were observed to be concentration- and time-dependent (Figure 4). In *S. flexneri* S1 strain, highest inhibition of 54.8% was recorded at 200 µg/mL at 48 h, while lowest reduction of 26.3% was observed at 50 µg/mL concentration of MK-AgNPs. Similarly, strains S2, S3, and S4 treated with 200 µg/mL of synthesized AgNPs resulted in highest inhibition of 54.1%, 58.6%, and 59.5%, respectively, at 48 h. Antimicrobial activity of *M. indica* against *Shigella* is well-documented [27,28,29,30]. The MIC of MK-AgNPs against four antibiotic-resistant *Shigella* strains was determined using microbroth dilution method in a 96-well microtiter plate. Furthermore, the antibacterial potential of MK-AgNPs was determined in terms of minimum inhibitory concentration (MIC), as depicted in Table S2. Synthesized AgNPs demonstrated MIC values of 20 µg/mL against *S. flexneri* (S1) and strain S4 while 10 µg/mL was recorded as MIC against strains S2 and S3. Omara et al. [31] reported MIC values of 16 μg/mL for chemically synthesized AgNPs against *Shigella* strains that were obtained from layer poultry farms.

**Figure 5:**
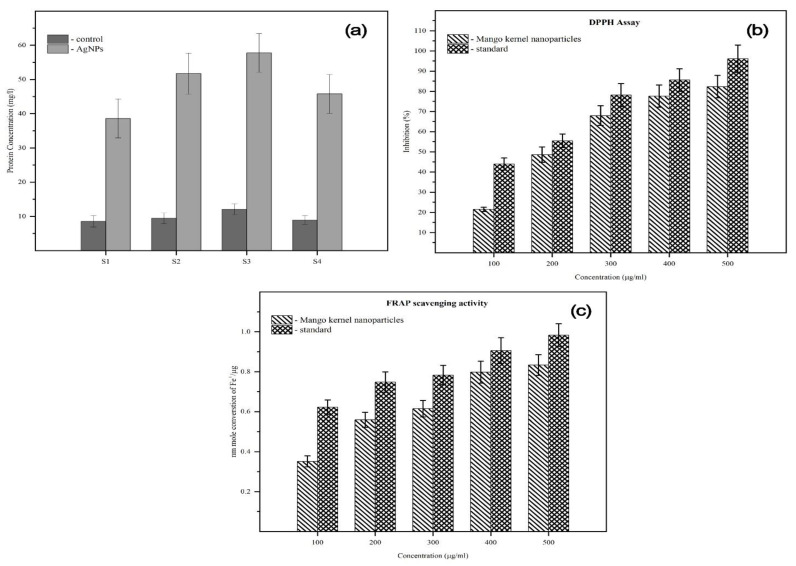
(**a**) Protein leakage assay. (**b**,**c**) DPPH and FRAP assay of MK-AgNPs.

Biosynthesized AgNPs act on the bacterial cells by disrupting the cell membrane, leading to subsequent protein leakage [18,32]. We analyzed the protein leakage in MK-AgNP-treated *Shigella* cells using Bradford assay. The results showed that the protein leakage in MK-AgNPs-treated *Shigella* isolates (S1, S2, S3, and S4) was recorded as 3.9, 5.2, 5.8, and 4.6 times higher compared to their respective controls (Figure 5a). Thus, it is envisaged that the bactericidal action of MK-AgNPs is due to the high infiltration of the bacterial cell wall by Ag, leading to release of the intracellular contents and cell death.

NAntioxidants are materials that neutralize the effects of reactive oxygen species (ROS) and prevent cell damage caused by them. There has been an enormous demand for antioxidants; in particular, natural antioxidants are in huge demand these days due to their ability to prevent diseases and to improve public health. Therefore, MK-AgNPs were tested for their scavenging of DPPH at concentrations ranging from 100–500 µg/mL. Concentration-dependent free radical scavenging activity was recorded as shown in Figure 5b. At 500 µg/mL, 82.5% of inhibition of DPPH was recorded as compared to the untreated control. Ascorbic acid was used as a standard. Additionally, FRAP assay was also performed to assess the antioxidant potential of scavenging activity of MK-AgNPs. As evident from Figure 5c, MK-AgNPs reduced 0.88 Fe^+^/µg whereas the standard (ascorbic acid) reduced 0.93 Fe^+^/µg at 500 µg/mL. Our findings are in line with those that reported AgNPs synthesized using aqueous extract of *M. indica* (seed). Photosynthesized AgNPs demonstrated dose-dependent antioxidant activity where DPPH activity of NPs exhibited an inhibition of 16–90% at tested concentrations (160–960 µg/mL) [23]. The results can be utilized for application of these NPs as antioxidants for various health benefits, especially against oxidative stress-related diseases and disorders.

## 3. Materials and Methods

### 3.1. Plant Sample Collection and Preparation

Ripe fruit seeds of *Mangifera indica* L. were collected from local market, juice shops, and food processing units in Erode, Tamil Nadu, India. The collected plant sample was authenticated by the Botanical Survey of India, Southern Regional Centre, and Coimbatore, Tamil Nadu, India. Mango seeds were washed with tap water and kernels were separated and then dried for 5 days at 40 °C and then 2 days at 50 °C. The kernel seeds were powdered for extraction.

### 3.2. Mango Seed Kernel Extract Preparation

The extraction process was carried out using a percolation method following the procedure described by Talaat et al. [33], with slight modifications. Briefly, 50 gm of kernel powder was extracted overnight with 250 mL of 70% ethanol in a shaking incubator (100 rpm) at room temperature. The extract was centrifuged for 15 min at 3500 rpm and the resulting supernatant was filtered through a Whatman No.1 filter paper. The filtrate (extract) was used for the green synthesis of AgNPs.

### 3.3. Synthesis of Mango Kernel-Based Silver Nanoparticles (MK-AgNPs)

Biosynthesis of MK-AgNPs was accomplished following the standard method described previously [32]. Briefly, 20 mL of ethanolic extract was added to 80 mL of 1 mM AgNO_3_ solution and stirred with a magnetic stirrer at room temperature. Silver nitrate was condensed to silver ions, and it was confirmed by change of colour from yellow to brown solution. The fully reduced solution was centrifuged at 5000 rpm for 30 min. After centrifugation, the nanoparticle pellets were separated and washed with ultra-pure water and finally lyophilized to obtain powder.

### 3.4. Characterization of MK-AgNPs

The properties of biosynthesized MK-AgNPs were analyzed using UV–Vis spectroscopy. Samples were diluted with ultra-pure water and then absorbance spectrum was recorded from 300 to 600 nm using UV–Vis spectrophotometer (Shimadzu UV-1800, Kyoto city, Japan). Functional group analysis was performed using Fourier Transform Infra-Red Spectroscopy (FTIR). Crystallinity, phase analysis, and purity were determined using electron X-ray diffractometry (XRD) at 40 kV and 30 mA using Cu-Ka radiation. Pellets were prepared using two milligrams of MK-AgNPs and 200 mg of KBr (IR grade) and were assessed by FTIR (Thermo-Nicolet-380 Model, Maddison, WI, USA) [34]. Particle shape and size were investigated using scanning electron microscopy (SEM). The particle size was further analyzed using particle size analyzer (PSA) spectroscopy (PSA LA2800B, Jinan, China). The biosynthesized MK-AgNPs were soaked in low-concentrated ethyl alcohol and ultra-sonicated for 10 min. After sonication, MK-AgNPs were subjected to 245 nm laser wavelength [17].

### 3.5. Phytochemical Analysis

The phytochemical analysis of kernel seeds for detection of various classes of active chemical constituents was achieved using standard protocols described previously [35].

### 3.6. Isolation of Shigella from Diarrheal Stool Samples

A total of 30 diarrheal stool samples were collected from the Microbiological Lab, Deepa Micro Lab in Erode, Tamil Nadu, India. The samples were processed and the *Shigella* isolates were identified according to Bergey’s Manual of Systematic Bacteriology through microscopy and biochemical test (IMViC). Further identification was performed using 16s rRNA partial sequencing, and the sequence was submitted to GENBANK.

### 3.7. Selection of Multidrug-Resistant Shigella Isolate

The drug resistance pattern of *Shigella* isolates was evaluated on Mueller–Hinton agar plates following the method described earlier [36]. Briefly, bacteria were grown in nutrient broth for 8 h and the turbidity was adjusted to McFarland standard (1 × 10^8^ CFU/mL). A sterile cotton swab was used to spread the bacterial suspension evenly over the entire surface of the Mueller–Hinton agar plate. Next, antibiotics were used to assess the sensitivity/resistance pattern among the isolated bacteria: Ampicillin (10 μg), Ciprofloxacin, (10 μg), Norfloxacin (10 μg), Amikacin (30 μg), Gentamicin (30 μg), Ceftriaxone (10 μg), Azithromycin (10 μg), Trimethoprim (10 μg), Tetracycline (30 μg), and Chloramphenicol (30 μg).

### 3.8. Antibacterial Activity of MK-AgNPs

Antibacterial activity of MK-AgNPs against isolated enteric bacteria was investigated using the method described by Ameen et al. [18]. Briefly, multidrug-resistant *Shigella* isolates designated as S1, S2, S3, and S4 were grown in the presence of various MK-AgNPs concentrations, such as 50, 100, 150, and 200 μg/mL. At fixed time intervals (0–48 h), the growth of bacteria was analyzed by recording the absorbance at 600 nm using UV–Vis spectrophotometer.

### 3.9. Minimum Inhibitory Concentration (MIC) of MK-AgNPs

The MICs of MK-AgNPs were investigated in a 96-well microtiter plate against four drug-resistant clinical *Shigella* sp., with resazurin indicator. Briefly, various concentrations of MK-AgNPs (0–100 μg/mL) in 100 μL of Muller–Hinton broth were amended in different wells, and then 10 μL (5 × 10^8^ CFU/mL) of *Shigella* sp. suspension was added; subsequently, 10 µL of resazurin solution was also added to each well. Phosphate-buffered saline (pH 7.4) was used as negative control. The microtiter plate was incubated for 24 h at 37 °C. After, incubation plates were observed for colour change [32].

### 3.10. Protein Leakage Assay

Protein leakage assay was carried out using the Bradford method [34]. Briefly, resistant clinical *Shigella* sp. (S1, S2, S3, and S4) cells were treated with MK-AgNPs in nutrient broth (100 μg/mL) and incubated at 37 °C. At 0 h and 3 h intervals, the mixture was centrifuged at (6200× *g*) for 10 min and the supernatant was collected. Protein concentration was estimated by adding 200 μL of supernatant from each sample solution to 800 μL of Bradford reagent and incubating for 10 min. After incubation, optical density was measured at 595 nm. Bovine serum albumin (BSA) was used as standard positive control [34].

### 3.11. Antioxidant Activity of MK-AgNPs

To determine the antioxidant potential of mango seed kernel extract, 1,1-Diphenyl-2-picrylhydrazyl (DPPH) method was used. Briefly, 0.1 mL ethanol extract and 0.9 mL of 0.1 mM DPPH (dissolved in ethanol) were incubated at room temperature for 30 min. Ascorbic acid was used as a standard and the absorbance values are measured at 517 nm [20].

DPPH scavenging, percent = [(O.D. control − O.D. sample)/O.D. control] × 10 (1)

### 3.12. Ferric Reducing of Antioxidant Power Assay (FRAP Assay)

Different concentrations of the ethanol extract of *M. indica* seed kernel (10–50 μg mL^−1^) were added to 2.5 mL of 0.2 M sodium phosphate buffer (pH 6.6) and 2.5 mL of 1% potassium ferric cyanide [K_3_Fe (CN)_6_] solution, and the mixture was vortexed well. The vortexed mixture was incubated for 20 min at 50 °C using vortex shaker. Then, 2.5 mL of (10%) trichloro acetic acid was added to the test sample and centrifuged for 10 min at 3000 rpm. The supernatant (2.5 mL) was mixed with 2.5 mL of deionized water and 0.5 mL of 0.1% ferric chloride. The coloured solution was read at 700 nm against the blank with ascorbic acid as a reference standard using spectrophotometer [37].

## 4. Conclusions

Even though an enormous amount of research about green synthesized nanomaterials and their applications in the medical field is available, novel and safe therapeutic agents are still needed to combat the threat of emerging drug-resistant infection causing bacterial pathogens. In this study, in agreement with the saying ‘good from waste’, we have synthesized AgNPs from *M. indica* seed kernel. The phytochemicals in the seed kernel act as reducing and stabilizing agents for the formation of MK-AgNPs. The green synthesized nanoparticles were spherical in shape with an average size of 42.6 nm. They showed dose-dependent antioxidant activity and exhibited substantial antibacterial activity towards isolated multidrug-resistant *Shigella* clinical isolates. Protein leakage assay indicated that the NPs disrupt the cell membrane, and this could plausibly be responsible for the effective anti-*Shigella* activity. The biosynthesized MK-AgNPs can be therapeutically used to control shigellosis and related diseases. In particular, in the less developed countries, these NPs could be a financially viable and sustainable alternative to treat infectious entero-pathogenic bacteria. Antioxidant activity demonstrated by the AgNPs could find application as natural antioxidants which can be exploited in the treatment of diseases caused due to excessive ROS productive and oxidative stress. Further work in understanding the molecular mechanism and in vivo studies are required.

## Data Availability

The data presented will be made available on request by the corresponding authors.

## Supplementary Materials

The following supporting information can be downloaded at: https://www.mdpi.com/article/10.3390/molecules28062468/s1, Figure S1: (a) and (b) Resistant profile clinical Shigella isolates; Table S1: Phytochemistry analysis of mango kernel ethanol extract; Table S2: MIC of MK-AgNPs MDR *Shigella* isolates.
