# Supplementary material for: Biogenic Preparation and Characterization of Silver Nanoparticles from Seed Kernel of Mangifera indica and Their Antibacterial Potential against Shigella spp
Source: Molecules. 2023 Mar 8;28(6):2468. doi: 10.3390/molecules28062468 (PMC10054846; doi:10.3390/molecules28062468)
Supplement: Supplementary file 1 [file molecules-28-02468-s001.zip › molecules-1979555-supplementary.pdf]

Supplementary material

Table S1 Phytochemistry analysis of mango kernel ethanol extract

**Biomolecules**	**Test**	**Result**	**Inference**
Phenols	$FeCl_2$	Bluish black colour	+
Saponins	Foam test	Foam persist for 15 min	-
Alkaloids	Mayer's test	White ppt	+
Flavonoids	Alkaline reagent	Yellow colour	+
Tannins	Ferric chloride	Green or violet ppt	+

Table S2 MIC of MK-AgNPs MDR *Shigella* isolates

**Resistant *Shigella* isolates**	**Concentrations of MK-AgNPs (µg /mL)**										
	**0 (control)**	**10**	**20**	**30**	**40**	**50**	**60**	**70**	**80**	**90**	**100**
S1	+	+	-	-	-	-	-	-	-	-	-
S2	+	-	-	-	-	-	-	-	-	-	-
S3	+	-	-	-	-	-	-	-	-	-	-
S4	+	+	-	-	-	-	-	-	-	-	-

+ indicates bacterial growth   - indicates no growth.

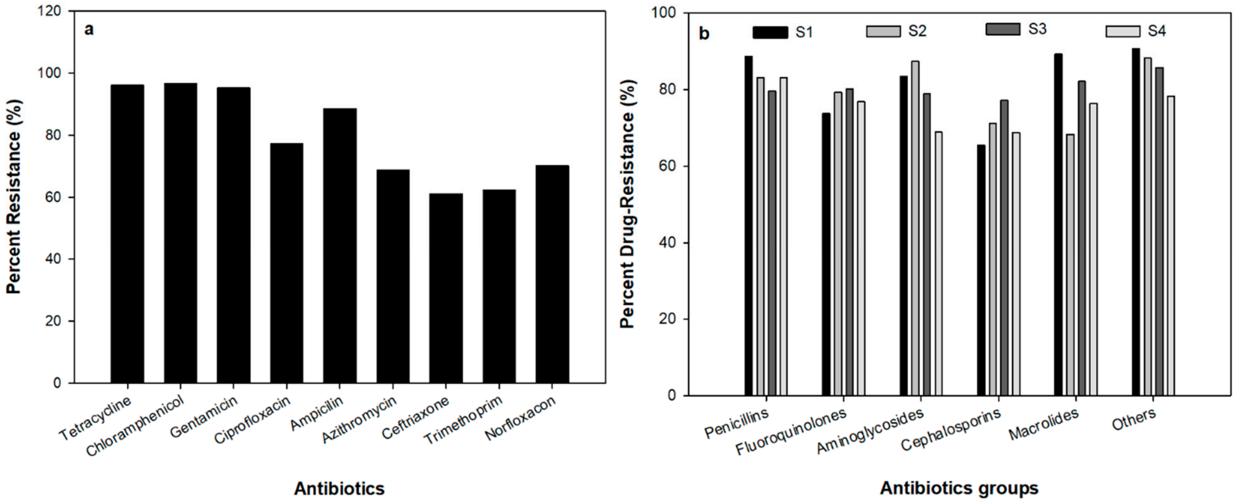

Figure S1 (a) and (b) Resistant profile clinical Shigella isolates
